# Evaluation of the Anticancer Activity of Phytomolecules Conjugated Gold Nanoparticles Synthesized by Aqueous Extracts of *Zingiber officinale* (Ginger) and *Nigella sativa* L. Seeds (Black Cumin)

**DOI:** 10.3390/ma14123368

**Published:** 2021-06-18

**Authors:** Alaa H. Alkhathlan, Hessah A. Al-Abdulkarim, Merajuddin Khan, Mujeeb Khan, Musaed Alkholief, Aws Alshamsan, Aliyah Almomen, Norah Albekairi, Hamad Z. Alkhathlan, M. Rafiq H. Siddiqui

**Affiliations:** 1 Department of Chemistry, College of Science, King Saud University, Riyadh 11451, Saudi Arabia; aalkhathlan1@ksu.edu.sa (A.H.A.); hessah@ksu.edu.sa (H.A.A.-A.); mkhan3@ksu.edu.sa (M.K); kmujeeb@ksu.edu.sa (M.K.); 2 Department of Pharmaceutics, College of Pharmacy, King Saud University, Riyadh 14511, Saudi Arabia; malkholief@ksu.edu.sa (M.A.); aalshamsan@ksu.edu.sa (A.A.); 3 Nanobiotechnology Unit, College of Pharmacy, King Saud University, Riyadh 14511, Saudi Arabia; alalmomen@ksu.edu.sa; 4 Department of Pharmaceutical Chemistry, College of Pharmacy, King Saud University, Riyadh 11451, Saudi Arabia; 5 Department of Pharmacology and Toxicology, College of Pharmacy, King Saud University, Riyadh 11451, Saudi Arabia; nalbekairi@ksu.edu.sa

**Keywords:** *Zingiber officinale*, *Nigella sativa*, plant extract, Au NPs, synthesis

## Abstract

The conventional physical and chemical synthetic methods for the preparation of metal nanoparticles have disadvantages as they use expensive equipment and hazardous chemicals which limit their applications for biomedical purposes, and are not environment friendly. However, for the synthesis of biocompatible nanomaterials, plant-based techniques are eco-friendly and easy to handle. Herein a simple, single-step biosynthesis of gold nanoparticles using aqueous extracts of *Nigella sativa* (NSE) and *Zingiber officinale* (GE) as a reducing and capping agent has been demonstrated. The formation of gold nanoparticles (Au NPs) was confirmed by X-ray diffraction, UV-Vis, and EDS spectroscopies. Spectroscopic and chromatographic analysis of GE and NSE revealed the presence of bioactive phytochemical constituents, such as gingerol, thymoquinone, etc., which successfully conjugated the surface of resulting Au NPs. TEM analysis indicated the formation of smaller-sized, less-aggregated, spherical-shaped Au NPs both in the case of GE (~9 nm) and NSE (~11 nm). To study the effect of the concentration of the extracts on the quality of resulting NPs and their anticancer properties, three different samples of Au NPs were prepared from each extract by varying the concentration of extracts while keeping the amount of precursor constant. In both cases, high-quality, spherical-shaped NPs were obtained, only at a high concentration of the extract, whereas at lower concentrations, larger-sized, irregular-shaped NPs were formed. Furthermore, the as-prepared Au NPs were evaluated for the anticancer properties against two different cell lines including MDA-MB-231 (breast cancer) and HCT 116 (colorectal cancer) cell lines. GE-conjugated Au NPs obtained by using a high concentration of the extract demonstrated superior anticancer properties when compared to NSE-conjugated counterparts.

## 1. Introduction

Currently, cancer is one of the major causes of death in the world, and according to WHO, the number of cancer patients will continue to increase at a higher pace, which may cause millions of deaths per year [1,2]. This disease has tremendously strained our medical facilities and heavily burdened health care professionals and scientists [3]. Thus, for the treatment of cancer, research activities are extensively focused on the development of alternative therapies and advanced technologies [4,5]. Among different types of therapies, chemotherapy is the most commonly applied method for the treatment of a variety of cancers, which involves a combination of various cytotoxic agents including antimetabolites, alkylating agents, and other biological compounds [6]. However, the majority of these cytotoxic agents are known to cause severe side effects, and their inappropriate use can potentially develop multidrug resistance (MDR) [7,8,9].

To deal with these problems, the development of novel antitumor agents based on various biologically active constituents such as natural dietary compounds, plant extracts, nanomaterials, and/or the synergistic combinations of different therapeutics have been encouraged for cancer therapy [10,11]. Particularly, chemotherapy based on natural products, including phytomolecules, has proved to be extremely effective due to its vast structural diversity [12]. Indeed, most of the currently applied anticancer drugs, such as vincristine, vinblastine, doxorubicin, etc., originated from natural products [13]. For several decades, plants are being screened for medicinal purposes, and their active phytoconstituents are successfully isolated as anticancer agents [14]. Extracts of a variety of plants have displayed anticancer and cytotoxic activities due to the presence of alkaloids, flavonoids, terpenoids, and other polyphenolic compounds, such as ursolic acid, oleanolic acids, kaempherol, quercetin, asmatrine, etc., which mostly exerted cytotoxic activities by inhibiting cancer cell growth [15].

Recently, nanotechnology has revolutionized cancer diagnosis and therapy and played crucial role in enhancing the safety and efficacy of existing antitumor drugs [16]. So far, a variety of nanomaterials including inorganic, polymeric, and bio-molecular nanoparticles (NPs) were tested in cancer therapy for both diagnostic and/or therapeutic purposes, because of their novel physicochemical properties [17]. Nowadays, to enhance the effectiveness of anticancer drugs, synergistic combinations of different therapeutics are often applied in chemotherapy [18]. In this approach, multiple therapeutic agents are simultaneously or sequentially delivered to obtain drug synergy, which reduces the overlapping toxicity and minimizes the chances of MDR [19]. Therefore, the combination of nanomaterials with potential anticancer phytoconstituents may lead to enhanced bioavailability, biocompatibility, and tumor specificity, and may reduce toxicity, due to the synergistic effect of combined materials [20]. Due to their large surface area and unique physicochemical properties, nanoparticles not only contribute to the cytotoxic activity of combined therapeutics, but also help to enhance the inherited cancer preventive properties of active phytochemical constituents [21].

The application of inorganic nanoparticles in cancer therapy is a growing research field due to the particles’ intrinsic antitumor effects which effectively inhibit the formation, development, and progression of tumors [22]. Particularly, metallic nanoparticles have gained more interest during the last few decades due to their unique properties including their highly reactive surfaces which effectively promote the induction of apoptosis through ROS generation, which is considered as an attractive cancer treatment mechanism [23]. Apart from cytotoxic effects, inorganic metal complexes have long been known for their cytostatic properties and have been extensively explored in cancer therapy [24,25]. In this regard, various metal complexes were applied against different types of cancer cells, which successfully inhibited tumor development and metastasis occurrence [26]. However, the cytostatic properties of inorganic metallic nanoparticles have been poorly explored so far. Nevertheless, various studies suggested that metallic NPs also exert a cytostatic effect, but the precise mechanisms of the inhibitory effect are still not clear [27]. Indeed, the unique physicochemical and surface properties of metallic NPs were exploited in preparing conjugates of metallic nanoparticles with cytostatic drugs to enhance the efficacy of chemotherapy based treatments [28]. For instance, in one study, polyvinylpyrrolidone (PVP)-modified Au NPs demonstrated enhanced cytostatic effects when compared to the pristine Au NPs [29]. In another study, among L-Methionine capped silver (Ag@LM NPs) and gold (Au@LM NPs) nanoparticles (NPs), during their treatment with lymphocytes, Ag@LM NPs accelerated the micronuclei incidence and suppressed the cell proliferation while Au@LM NPs promoted cell proliferation, with no significant effects on micronuclei formation.

Among metal nanoparticles, gold nanoparticles are more promising because of their special properties, including surface plasmon resonance (SPR) and their strong ability of binding thiol and amine groups, which can be exploited to customize their surfaces for advance applications [30]. Indeed, Au NPs are known to exhibit efficient anti-tumor activities through the induction of oxidative stress, which is confirmed by both in vivo and in vitro cytotoxic studies [31]. However, studies have suggested that in certain cases, the direct contact of naked NPs with healthy tissues may lead to their destruction, which can be minimized by the encapsulation or functionalization of nanoparticles [32,33]. The encapsulation of NPs is typically performed by various surface functionalization methods using different types of ligands including biomolecules and a variety of phytoconstituents [34].

Typically, Au NPs are prepared via various synthetic routes including chemical, sonochemical, or photochemical methods, which require high temperature and pressure, harsh chemicals, and hazardous reducing agents [35]. Additionally, in most cases external stabilizers are required to prevent the agglomeration or further growth of the particles [36]. However, due to environmental issues and considering the importance of Au NPs in biomedical applications, the development of eco-friendly protocols using non-toxic reagents under ambient conditions are highly attractive [37]. The application of sustainable processes using renewable materials leads to the synthesis of cost-effective, bio-compatible Au NPs [38]. Green synthesis of Au NPs and their hybrid nanocomposites is typically achieved using a variety of renewable materials including biomolecules, biopolymers, microorganisms, and plant extracts [39,40,41,42,43]. Using plant extracts as reducing and stabilizing agents, Au NPs with varied shapes have been prepared, such as spherical- and triangular-shaped Au; Ag and Au–Ag bimetallic alloy NPs were prepared by using *Pulicaria undulata* (L.) plant extract [44,45]. Plant extracts consist of different types of bioactive phytoconstituents such as polyphenols, terpenoids, phenolic acids, etc., with high structural diversity and efficient reducing abilities [46]. Thus, these types of bio-materials are strongly capable of reducing inorganic precursors to produce high-quality nanoparticles in a single-pot reaction [47]. Besides, bioactive phytoconstituents can be potentially adsorbed on the surface of resulting NPs during the synthesis, which may be crucial for their biomedical applications including anticancer therapy [48]. In this regard, we previously reported the synthesis of silver NPs using aqueous extracts of *Zingiber offcinale* (ginger) and *Nigella sativa* L. seeds (black cumin) [49]. The bio-active constituents of both extracts successfully reduced the silver precursor, and the residual phytomolecules were also adsorbed on the surface of resulting Ag NPs. The ginger extract is known to exhibit anticancer properties due to the presence of certain pungent vallinoids, such as [6]-gingerol and [6]-paradol, as well as some other constituents such as shogaols, zingerone, etc. [50]. Moreover, thymoquinone, α-hederin, thymol, thymohydroquinone, and carvacrol, etc., of *N. sativa* have demonstrated anticancer and cytotoxic activities [51].

**Scheme 1:**
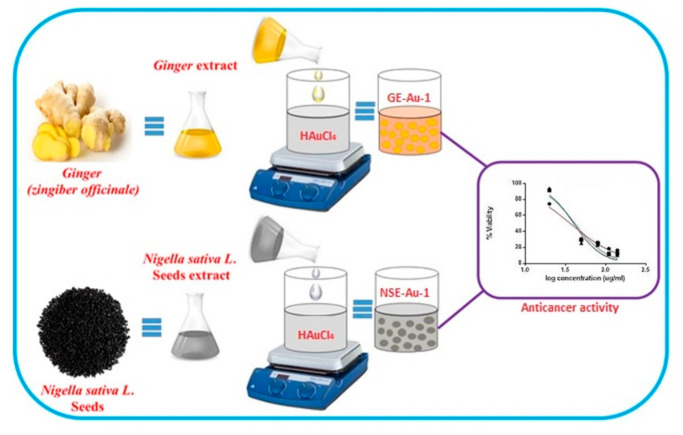
Graphical representation of gold nanoparticles prepared using NSE extract and (b) GE extract.

In this report, we prepared gold nanoparticles by the reduction of the gold ions using aqueous extracts of both *N. sativa* L. seeds (Black Cumin) and *Z. officinale* (ginger) (cf. Scheme 1). We investigated the effect of the concentrations of the reactant (chloroauric acid and plant extract) on the particle size and morphology. The Au NPs were characterized by using X-ray Diffraction (XRD), Energy Dispersive X-ray (EDS), and Transmission Electron Microscopy (TEM). In addition, the anticancer properties of phytoconstituents’ conjugated Au NPs obtained from both extracts were studied against breast cancer and colorectal cancer.

## 2. Materials and Methods

### 2.1. Materials

The plant materials were purchased from local stores in Riyadh, Saudi Arabia; chloroauric acid (HAuCl_4_; 99.999%) was purchased from Sigma-Aldrich Co., St. Louis, MO, USA.

### 2.2. Preparation of Plant Extracts

#### 2.2.1. *N. sativa*

The seeds of *N. sativa* (black seeds) (300 g) were grinded and boiled with 800 mL of deionized water and refluxed for 3 h. The aqueous solution was filtered and dried under reduced pressure in a rotary evaporator to give a dark color residue (21.9 g).

#### 2.2.2. *Z. officinale*

Fresh *Z. officinale* (ginger) (600 g) rhizomes were washed and chopped to small pieces and soaked in deionized water (1000 mL) and refluxed for 3 h. Then, they were filtered and dried under reduced pressure in a rotary evaporator to give a light brown color residue (5.7 g). An amount of 1 g/10 mL of each plant extract separately was used for the synthesis of gold nanoparticles.

### 2.3. Synthesis of Gold Nanoparticles

#### Gold with *Z. officinale* (GE) and *N. sativa* (NSE)

The reaction mixture was prepared by adding 10 mL of each plant extract separately to 90 mL of 10 mM HAuCl_4_ solution. The reaction was kept for 3 h at 90 °C to form the nanoparticles, which was indicated by the color change from light yellow to reddish brown. The mixture was centrifuged at 9000 rpm and washed with distilled water. In order to study the effect of the concentration of plant materials on the synthesis of Au NPs, three different samples were prepared from each extract by varying the concentration of plant material, while keeping the amount of gold precursor and temperature constant. For instance, three Au samples prepared by using *N. sativa* (NSE) were named as NSE-Au-1 (5 mL extract), NSE-Au-2 (10 mL extract), and NSE-Au-3 (20 mL extract), whereas in all these samples, ~1 mmol of gold precursor (HAuCl_4_) was used. Similarly, in the case of *Z. officinale* (GE), the samples were named as GE-Au-1 (5 mL), GE-Au-2 (10 mL), and GE-Au-2 (20 mL). In all experiments, the temperature was kept at 90 °C for 3 h.

### 2.4. Characterization

The optimal measurement of the synthesized Au NPs was recorded at different time intervals by the UV-1800 UV-Vis spectrophotometer (Shimodzo, Kyoto, Japan). It was performed in quartz cuvettes, and the scale of the wavelength was set between 200 and 800 nm. X-ray powder diffraction (XRD) was measured with Cu K radiation (λ = 1.5418 Å), Ultima IV X-ray (Rigaku, Tokyo, Japan). High-resolution transmission electron microscopy (HRTEM) and Energy-dispersive X-ray spectroscopy (EDX) (JEM 2100F (JEOL, Tokyo, Japan)) were used. The accelerating voltage used for TEM measurements is 200 kV.

### 2.5. Methodology

#### Cytotoxic Assessment of *Z. officinale* and *N. sativa* Doped AU-NPs

The cytotoxicity of Au-NPs doped with different concentrations of ginger and *N. sativa* extracts were examined against MDA-MB-231 (breast cancer) and HCT 116 (colorectal cancer) cell lines. Cells were seeded in a 96-well culture plate with a density of 1 × 10^4^ cells/well in 100 µL culture media and incubated for 24 h at 37 °C. Cells were then treated with the prepared nanoparticles in various concentrations of 20, 50, 80, 110, and 140 µg/mL and incubated for 24, 48, and 72 h. Twenty microliters of 2.5 mg/mL of 3-(4,5-dimethylthiazol-2-yl)-2,5-diphenyltetrazolium bromide (MTT) in PBS was added to cells, and cells were further incubated for 4 h at 37 °C. MTT solutions were then completely removed, and 100 μL DMSO was added to solubilize formazan crystals. The absorbance was detected at 590 nm using the Spectramax 250 microplate reader (molecular device, San Jose, CA, USA), and viability (%) was calculated as (optical density (OD) of treated group/OD of control group) × 100.

## 3. Results and Discussion

**Figure 1:**
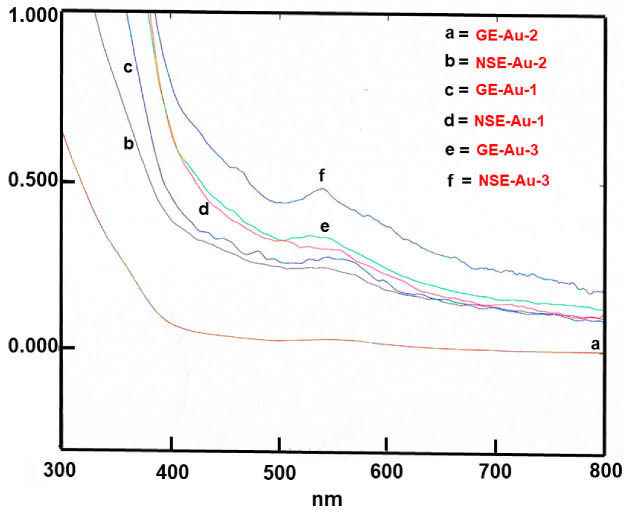
UV-Vis absorption spectra of gold nanoparticles prepared from both GE (5, 10, and 20 mL) and NSE (5, 10, 20 mL) at different concentrations of extracts.

In this report, gold nanoparticles (Au NPs) were prepared using the *N. sativa* extract (NSE) and ginger extract (GE). The reactions were performed under facile conditions using an aqueous solution of respective precursor salts HAuCl_4_ and GE or NSE extracts without the addition of any other external reducing agent. A total six different samples were prepared using different concentration of extracts. For instance, three samples were synthesized with varied concentrations of NSE which were designated as NSE-Au-1 (5 mL extract), NSE-Au-2 (10 mL extract), and NSE-Au-3 (20 mL extract). Three more samples were prepared by using different concentrations of GE, and these samples are named as GE-Au-1 (5 mL), GE-Au-2 (10 mL), and GE-Au-2 (20 mL). In all experiments, the temperature was kept at 90 °C for 3 h, whereas the concentration of gold precursor was kept constant in all the reactions. The color of the aqueous solution of HAuCl_4_ gradually changed from light yellow to dark pink upon the addition of extracts (GE and NSE) while stirring for several hours (~3 h). This indicated the formation of Au NPs, which is further confirmed by UV-Analysis due to the appearance of the surface plasmon resonance of Au NPs. Figure 1 displays the UV-Vis spectra of the synthesized Au NPs at 90 °C by using both NSE and GE. In both cases of NSE and GE, a lower concentration of plant extract did not yield good quality of nanoparticles, whereas the appearance of sharp absorption bands at ~540 nm indicates the formation of spherical-shaped Au NPs (cf. Figure 1). This is in accordance with the previously reported studies, which revealed that a sharper absorption at a higher wavelength indicates the formation of spherical-shaped gold nanoparticles [52]. For instance, in the case of honey-mediated synthesis of Au NPs, with the increasing concentration of honey, the UV peaks gradually changed from a broad peak at a lower wavelength to a sharp band at a higher wavelength which is associated with the formation of spherical-shaped gold nanoparticles [53].

**Figure 2:**
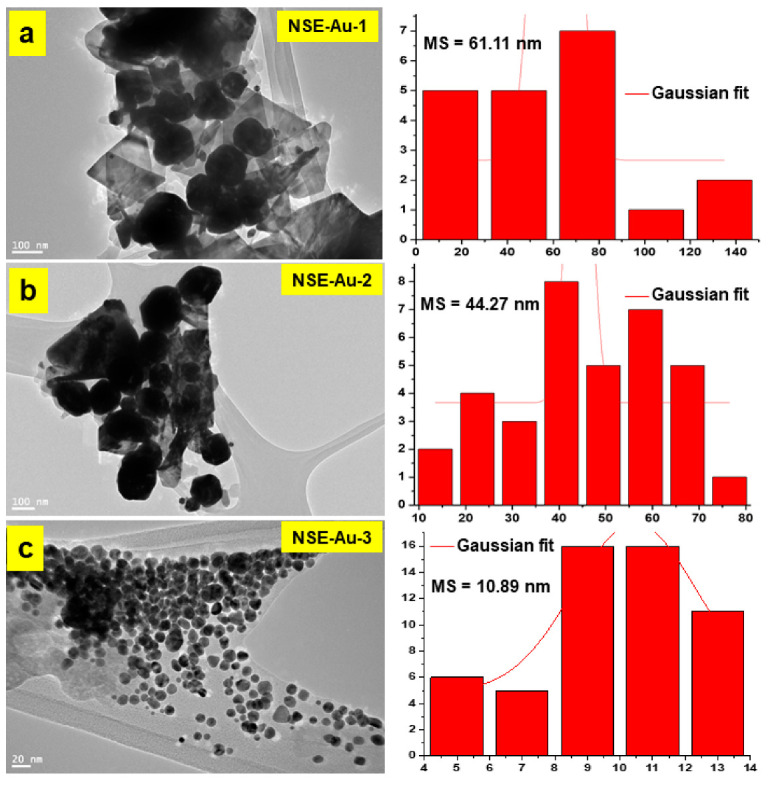
TEM images of Au NPs prepared by using different concentrations of NSE extract, (**a**) Au NPs prepared by using 5 mL of NSE and their size distribution, (**b**) NPs obtained by using 10 mL NSE, and (**c**) Au NPs obtained by using 20 mL of NSE extract and their size distribution. * MS = Mean Size, red line represents Gaussian fit.

The size and morphology of resultant Au NPs prepared with different concentrations of NSE and GE were evaluated by transmission electron microscopy (TEM). Figure 2 shows an overview of the green-synthesized Au NPs using different concentrations of the NSE extract. Notably, at a lower concentration of NSE, both at 5 and 10 mL of NSE, large-sized, irregular-shaped, agglomerated Au NPs were formed as shown in Figure 2a,b. NPs with a size range between 10 and 150 nm, with a mean size of 61.11 nm, were obtained in the case of NSE-Au-1 which was prepared by using a low concentration of 5 mL NSE. Upon doubling the concentration to 10 mL (NSE-Au-2), the average size of the NPs slightly decreased to 44.27 nm as shown in Figure 2b. However, when the concentration of NSE was further increased to 20 mL, small-sized, spherical-shaped, nicely distributed Au NPs were obtained with a size range of 5 to 15 nm, with a mean size of 10.89 nm, as shown in Figure 2c.

**Figure 3:**
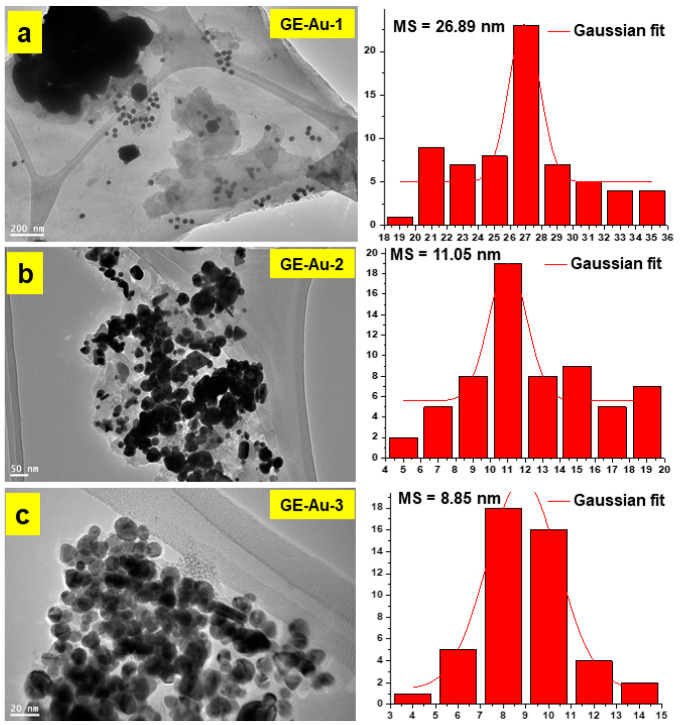
TEM images of Au NPs prepared by using different concentrations of GE extract, (**a**) Au NPs prepared by using 5 mL of GE and their size distribution, (**b**) NPs obtained by using 10 mL GE, and (**c**) Au NPs obtained by using 20 mL of GE extract and their size distribution. * MS = Mean Size, red line represents Gaussian fit.

Similarly, the concentration of ginger extract (GE) also slightly affected the size and agglomeration of the resultant NPs; however, the effect is not so obvious as compared to the NSE extract. Nevertheless, in this case, a higher concentration of GE produced spherical-shaped, smaller-sized Au NPs. For example, upon using a lower concentration of GE, larger-sized, irregular-shaped NPs were obtained with an average size distribution of 26.89 nm, whereas when the amount of GE was doubled to 10 mL, the size of NPs was reduced to less than half, to an average size of 11.05 nm, as shown in Figure 3a,b, respectively. However, in both cases, irregular-shaped, agglomerated nanoparticles were formed. Upon further increasing the GE amount to 20 mL, smaller-sized, spherical-shaped Au NPs were formed which are less aggregated when compared to the NPs obtained at a lower concentration of GE, as shown is Figure 3c. The elemental compositions of the green-synthesized samples were also determined by EDS (Energy Dispersive X-ray Spectrometry). The EDS of Au NPs obtained by using a high concentration of extracts (20 mL) of both NSE (NSE-Au-3, cf. Figure S1) and GE (GE-Au-3, cf. Figure S2) are provided in the Supplementary Information. The spectra reveal a clear elemental composition of the green-synthesized AuNPs. Figure S1 demonstrates the EDS signals of Au samples, exhibiting an intense signal at ~2 keV, which strongly suggests the presence of Au as the major element in the sample, since Au has an optical absorption in this range due to the SPR [54] (Su. Notably, increasing and decreasing the concentration of plant extract did not have any effect on the synthesis of nanoparticles. Au NPs have been formed even with a lower amount of plant extract (5 mL), as indicated by the EDS spectra of NSE-Au-1, NSE-Au-2, GE-Au-1, and GE-Au-2 (data not shown).

**Figure 4:**
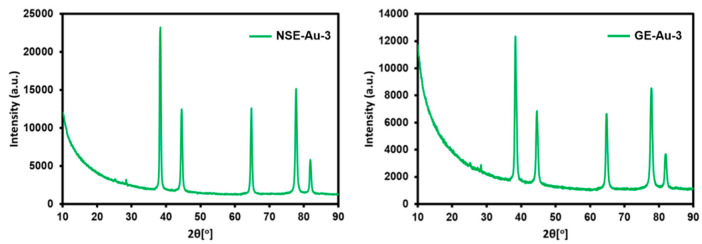
XRD pattern of gold nanoparticles (Au NPs) prepared by a using high concentration of both NSE and GE extract (20 mL in each case).

In addition, the formation of Au NPs was also confirmed by XRD analysis which also highlighted the crystalline nature of the biosynthesized NPs. Figure 4 displays the representative XRD pattern of Au NPs synthesized by using both NSE and GE using a high concentration of extract (20 mL) In both spectra (NSE-Au-3 and GE-Au-3), five distinct diffraction peaks were observed at about 2θ = 38.2, 44.6, 64.9, 77.8, and 81.7, which can be indexed as the (111), (200), (220), (311), and (222) lattice planes, belonging to the standard face-centered cubic phase (*fcc*) of metallic gold, as shown in Figure 4 (Shi et al., 2015). This clearly indicates the formation of crystalline Au NPs. Notably, no additional peaks are observed in the XRD spectra of Au NPs; this revealed the high purity of the formed Au NPs. The diffraction peak corresponding to the (111) phase at 2θ = 38.2 was overwhelmingly stronger than the rest of the peaks, suggesting that (111) was the primary orientation [55]. The XRD patterns of the remaining Au NPs samples prepared by using a low concentration of both NSE and GE extracts, including NSE-Au-1 and 2 (cf. Figure S3) and GE-Au-1 and 2 (cf. Figure S4), are provided in the Supplementary Information.

**Figure 5:**
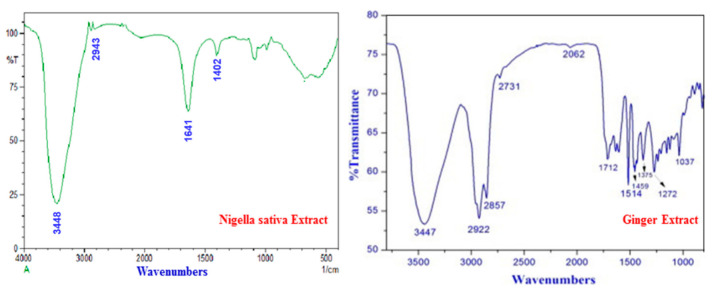
FT-IR spectra of pure *N. sativa* (NSE) and *Z. officinale* (GE) Extract.

The dual role of the NSE and GE as a bio-reductant and capping agent was confirmed by FT-IR analysis of the as-prepared Au NPs. Plant extracts are typically known to possess various active phytoconstituents which possess effective reduction potential such as flavonoids, polyphenols, saponins, terpenes, etc. [56]. The FTIR spectrum of the GE confirms the presence of various types of polyphenols as shown in Figure 5. For instance, the presence of a strong broad band at 3447 cm^−1^ can be attributed to (-OH) stretching vibrations. The presence of bands with strong to medium intensities were also observed at 2922 cm^−1^ and 2857 cm^−1^ which was assigned as carboxylic acid groups. Other strong to medium intensity bands were also observed at 2731 cm^−1^ due to the presence of aldehydes and at 1712 cm^−1^ indicated the presence of C=O groups. Some other bands appeared at 1636 cm^−1^ and 1609 cm^−1^; 1514 cm^−1^ and 1459 cm^−1^; 1375 cm^−1^; 1272 cm^−1^; 1159 cm^−1^; 1037 cm^−1^; and 618 cm^−1^, which might be due to the presence of CH_2_=CH_2_; aromatic C=C; alkene; methyl -CH_3_; ether; alcohol; and phenols, respectively. Generally, free radical scavenging and antioxidant activity of phenolics (e.g., flavonoids, phenolic acids) mainly depend on the number and positions of hydrogen-donating OH groups on the aromatic rings of the phenolic molecules [57].

**Figure 6:**
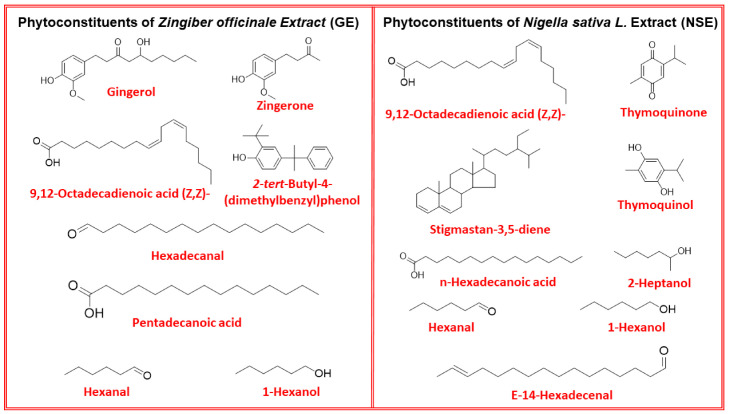
The presence of some of the different types of hydroxyl group containing phytochemical constituents of *Z. officinale* (GE) and *N. sativa* (NSE) extract.

Similarly, the IR spectrum of NSE also strongly indicated towards the presence of flavonoids and phenolic compounds, due to the existence of various absorption peaks belonging to the alcoholic, carboxylic, ester, and ether groups (cf. Figure 5), such as the absorption peaks at 3448 cm^−1^ corresponding to the hydrogen-bonded hydroxyl (OH), and the peak at 2943 cm^−1^ indicates the presence of C−H. The absorption peaks situated around 1641 and 1402 cm^−1^ are the characteristic peaks for the C=O and C=C stretching, respectively, of the aromatics. Notably, most of the FT-IR spectra of green-synthesized NPs are almost similar to the FT-IR spectrum of the respective pure extracts (such as NSE and GE; data not shown), except slight minimal shifts in a few peaks. This striking resemblance between these two spectra clearly suggests that some of the residual phytomolecules of the extracts remained attached on the surface of the green-synthesized Au NPs. Indeed, the presence of polyphenolic compounds in the extracts of both NSE and GE is also confirmed by GC/MS analysis. Figure 6 depicts the presence of some of hydroxyl groups containing phytoconstituents of both NSE and GE extracts. The ginger extract consists of gingerol as the major compound, apart from various others including zingerone, pentane decanoic acid, hexanal, etc. The NSE extract contains thymoquinone, thymoquinol, *n*-Hexadecanoic acid, and so on. The details of all the compounds detected in the extracts of both GE and NSE are provided in the Table 1.

### Cytotoxicity Study

The application of naturally-existing agents to regulate tumorigenesis is increasing rapidly. So far, a variety of natural products, including herbal extracts, pure plant-based active constituents, and food additives have been extensively studied, and most of them exhibited excellent anticancer properties and cancer-ameliorating effects. In this regard, the wide-range anticancer properties of *Z. officinale* (GE) and *N. sativa* (NSE) extract, which are commonly known as ginger and black seed, respectively, have been excessively studied using both in vitro and in vivo models [58,59]. The anticancer properties of ginger extract (GE) is mainly attributed to the presence of some pungent constituents such as vallinoids, viz. [6]-gingerol, and [6]-paradol, as well as some other constituents such as shogaols, zingerone, etc. [50], whereas most of the anticancer properties of NSE have been commonly associated with its major constituent, thymoquinone (TQ), which possesses anti-proliferative, pro-apoptotic, anti-oxidant, anti-mutagenic, anti-angiogenic, and anti-metastatic effects on different types of cancer cells [51]. In this study, extracts of NSE and GE were used to prepare Au NPs. The active phytochemical constituents of these extracts, including gingerol, thymoquinone, etc., which are present in both NSE and GE, as confirmed by various spectroscopic and chromatographic techniques, are known to possess reducing and stabilizing properties [60,61]. Notably, these biologically active phytoconstituents conjugated to the surface of resulting Au NPs which may exert a potential cytotoxic effect on the NPs. To confirm this, the anticancer properties of Au NPs prepared by using different concentrations of NSE (NSE-Au-1,2,3) and GE (GE-Au-1,2,3) extracts are studied to evaluate the effect of the concentration of plant extract on the biological properties of resulting nanoparticles.

**Figure 7:**
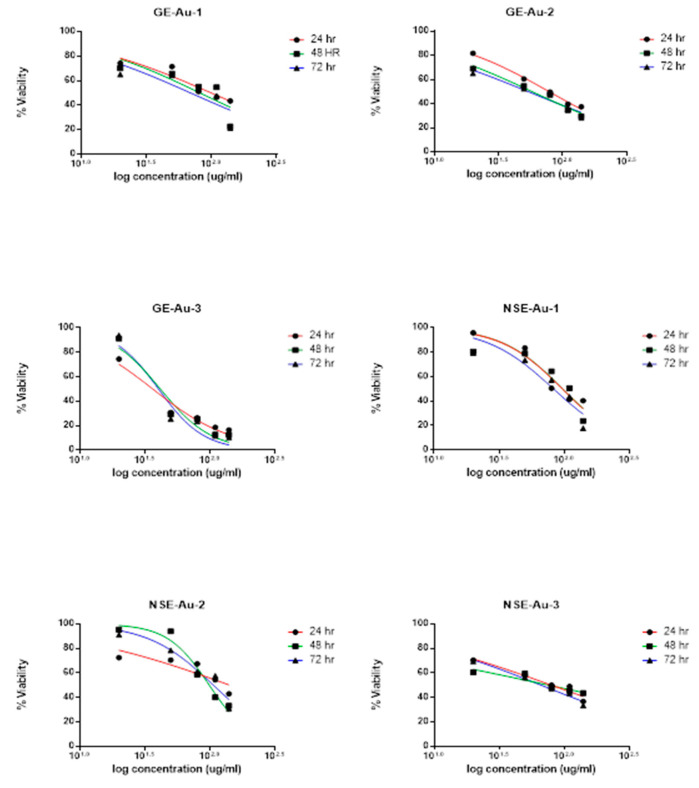
Cytotoxicity of various concentrations of GE- and NSE-conjugated Au-NPs against MDA-MB-231 cells. Results are shown as mean ± SD (*n* = 3).

The cytotoxicity of GE- and NSE-conjugated Au-NPs were evaluated in MDA-MB-231 (breast cancer) and HCT 116 (colorectal cancer) cell lines. Results showed that the majority of the Au samples tested, which were prepared by different concentrations of extracts, were able to decrease the viability of MDA-MB-231 cells in a dose-dependent manner. In the case of MDA-MB-231 breast cancer cell lines, among all these samples, Au NPs prepared with the high concentration of GE, such as 20 mL (GE-Au-3) and 10 mL (GE-Au-2), exhibited the lowest IC50s of 40.3 ± 0.1 µg/mL and 54 ± 0.13 µg/mL, respectively (Table 2), and maximum viabilities at 72 h and 140 ug/mL of about 10% and 28%, respectively. Primarily, GE-Au-3 was not as effective in inducing cytotoxicity in the lowest concentration tested, 20 µg/mL, where the viability increased from about 74% at 24 h to about 93% at 72 h, whereas, all the Au samples prepared from NSE did not show any striking cytotoxicity against MDA-MB-231 breast cancer cell lines, when compared to the samples synthesized by GE. For instance, the lowest IC50 value of 66.67 ± 0.4 was obtained in the case of NSE-Au-3, which was prepared by using the highest concentration of NSE extract (20 mL). In the case of samples prepared in lower concentrations of NSE, such as NSE-Au-1 and NSE-Au-2, IC50 values of 106.7 ± 0.2 and 83 ± 0.3 µg/mL were obtained, respectively. This means that NSE-Au-1 showed a maximum viability of only 17.7% at 72 h and 140 µg/mL, whereas NSE-Au-2 demonstrated maximum viability of 31.1% with 140 µg/mL at 72 h. Although Au NPs samples prepared from low concentration of NSE showed an initial reduction in viability at 24 h, MDA-MB-123 cells started to acquire resistance to the formulation at lower concentrations, and only higher concentrations were able to exhibit effective reduction in cells’ viability (Figure 7).

**Figure 8:**
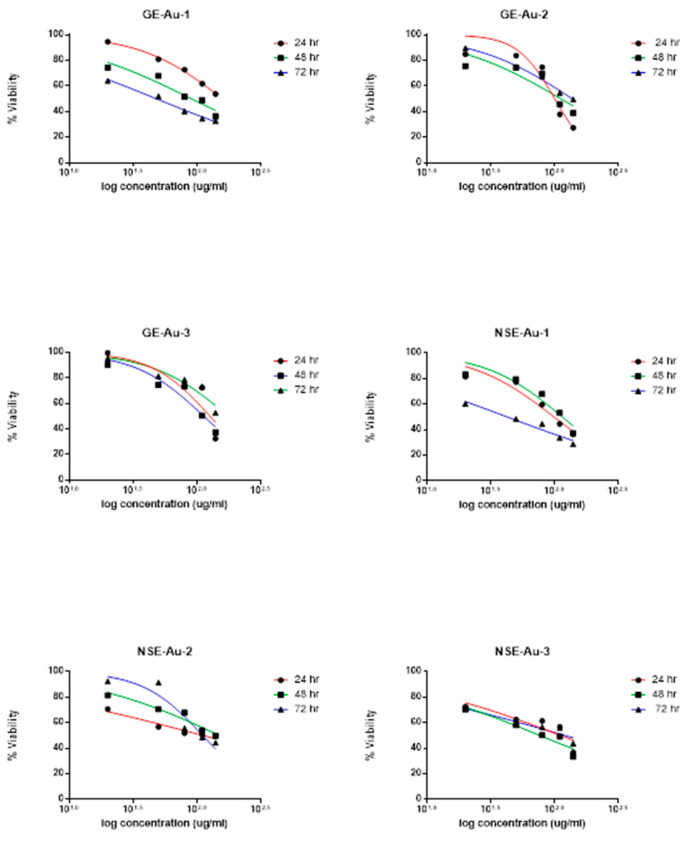
Cytotoxicity of various concentrations of GE- and NSE-conjugated Au-NPs against the HCT 116 cancer cell lines at different time points. Results are shown as mean ± SD (*n* = 3).

A similar trend of a dose-dependent decrease in viability was found with HCT 116 cells (Figure 8). Besides the dose-dependent decrease in viability, AuNPs synthesized from a high concentration of GE (20 mL, GE-Au-3) also showed an obvious time-dependent reduction in viability with IC50s reaching around 47.56 ± 0.21 µg/mL at 72 h (Table 3), whereas at a lower concentration of GE, i.e., at 5 mL (GE-Au-1) and 10 mL (GE-Au-2), negligible cytotoxicity was observed. In the case of NSE, the sample prepared with a high concentration of NSE (NSE-Au-3), on the other hand, showed an increase in viability at 48 h after an initial reduction in viability found at 24 hr. However, at 72 hr NSE-Au-3 exhibited a drastic reduction in viability (42.6 ± 0.11) which might infer that cells underwent a state of cytostasis before suffering from cytotoxicity [62]. It is also apparent that HCT 116 cells started to acquire resistance to NSE-Au-1 and NSE-Au-2 especially at lower concentrations at 48 and 72 h. On the contrary, cells treated with GE-Au-2 showed an increase in viability with even a higher formulation of concentrations at 72 h which might indicate cells’ resistance to treatment.

In the case of both MDA-MB-231 breast cancer cell lines and HCT 116 colorectal cancer cell lines, comparatively, the GE-conjugated Au NPs have demonstrated superior cytotoxicity than the NSE-stabilized samples. One possible reason could be the application of water extracts for the preparation of the samples in this study. It is noteworthy that the aqueous extract of ginger possesses considerable anticancer properties when compared to the aqueous fraction of NSE [63]. Notably, the bulk of the anticancer activity of NSE is mainly attributed to its quinone contents, including thymoquinone and its derivatives which are mainly present in the volatile and non-volatile oils of NSE [64], whereas the cytotoxicity of GE is associated with its water-soluble pungent vallinoids, such as gingerol, etc. Moreover, the high cytotoxicity of the GE-conjugated samples could also be associated with gingerol, which is detected as a major component in the GE (cf. Table 1). Hydroxyl groups present in bioactive gingerol may have facilitated the proper binding of this compound to the surface of Au NPs. Such cytotoxic phytoconstituents may have contributed to the anticancer properties of resulting Au NPs. Indeed, this is also indicated by the increasing cytotoxicity of the sample with increasing the concentration of plant extract, which ultimately led to the presence of more bioactive components on the surface of resulting NPs. In the case of NSE, possibly the Au NPs may have been stabilized by less-active, hydroxyl-containing phytoconstituents, such as thymoquinol, heptanol, hexanol, etc., which possess lower cytotoxicity when compared to highly active thymoquinone. However, thymoquinone may not have binded to the surface of Au NPs due to the absence of hydroxyl groups.

## 4. Conclusions

*Z. officinale* (GE)- and *N. sativa* L. seeds (NSE)-mediated green synthesis of Au NPs presented in this study offers an economically viable and facile approach for the preparation of biocompatible nanomaterials. Aqueous extracts of both GE and NSE demonstrated a concentration-dependent ability to produce high-quality, spherical-shaped, smaller-sized Au NPs. Bioactive phytochemical constituents of these extracts including gingerol, thymoquinol, heptanol, and hexanol successfully conjugated the surface of resulting NPs to deliver stabilized Au samples. Moreover, Au NPs prepared with a high concentration of both GE and NSE exhibited good cytotoxicity against MDA-MB-231 (breast cancer) and HCT 116 (colorectal cancer) cell lines. Comparatively, GE-conjugated Au samples exhibited superior cytotoxicity compared to the NSE-stabilized samples, which can be attributed to the presence of cytotoxic phytoconstituents in the aqueous extract of GE.

## Figures and Tables

**Table 1:** Identified chemical compounds from *N. sativa* and *Z. officinale*.

Peak	Compounds	RT (Min.)	MF	MW	Peak Area (%)	
					NS	ZO
1	Hexanal	6.28	C_6_H_12_O	100.16	0.4	4.8
2	1-Hexanol	8.43	C_6_H_14_O	102.17	0.7	0.8
3	2-Heptanol	9.09	C_7_H_16_O	116.2	0.3	-
4	Dodecane	20.80	C_12_H_26_	170.33	0.5	0.8
5	Tridecane	24.24	C_13_H_28_	184.36	-	0.6
6	Tetradecane	27.49	C_14_H_30_	198.39	0.6	1.0
7	Thymoquinone	28.81	C_10_H_12_O_2_	164.2	3.3	-
8	Thymoquinol	32.19	C_10_H_14_O_2_	166.22	0.5	-
9	Hexadecane	33.45	C_16_H_34_	226.44	-	1.0
10	Zingerone	34.87	C_11_H_14_O_3_	194.22	-	3.0
11	Hexadecanal	39.35	C_16_H_32_O	240.42	-	1.1
12	Pentadecanoic acid	40.64	C_15_H_30_O_2_	242.39	-	0.6
13	*n*-Hexadecanoic acid	42.84	C_16_H_32_O_2_	256.42	3.1	-
14	*2-tert*-Butyl-4-(dimethylbenzyl)phenol	46.74	C_19_H_24_O	268.4	-	1.0
15	(*Z*,*Z*)-9,12-Octadecadienoic acid	46.87	C_18_H_32_O_2_	280.45	11.4	1.0
16	3-(6-Hydroxy-3,7-dimethyl-octa-2,7-dienyl)-4-methoxy-phenol	50.56	C_17_H_24_O_3_	276.00	-	7.0
17	Gingerol	52.32	C_17_H_26_O_4_	294.38	-	11.3
18	(*E*)-14-Hexadecenal	52.35	C_16_H_30_O	238.41	0.7	-
19	Pentacosane	55.65	C_25_H_52_	352.68	-	1.5
20	*n*-Hexacosane	58.04	C_26_H_54_	366.70	0.8	-
21	*n*-Heptacosane	60.48	C_27_H_56_	380.73	1.3	-
22	Stigmastan-3,5-diene	61.40	C_29_H_48_	396.69	16.4	-

MF = Molecular formula; MW = Molecular weight; RT = Retention time; NS = *N. sativa*; ZO = *Z. officinale*.

**Table 2:** IC50 values (µg/mL) of various concentrations of GE- and NSE-conjugated Au-NPs against MDA-MB-231 breast cancer cell lines at different times.

Au-NPs	24 h	48 h	72 h
GE-Au-1	100.22 ± 0.38	82.26 ± 0.28	69.67 ± 0.25
GE-Au-2	78.85 ± 0.27	58.23 ± 0.07	54 ± 0.13
GE-Au-3	36.4 ± 0.12	41.76 ± 0.07	40.3 ± 0.1
NSE-Au-1	125.4 ± 0.21	99.04 ± 0.43	106.7 ± 0.22
NSE-Au-2	97.07 ± 0.11	97.76 ± 0.22	83 ± 0.3
NSE-Au-3	80.28 ± 0.21	76.54 ± 1	66.67 ± 0.42

**Table 3:** IC50 values (µg/mL) of various concentrations of GE- and NSE-conjugated Au-NPs against HCT 116 (colorectal cancer) cell lines at times.

Au-NPs	24 h	48 h	72 h
GE-Au-1	126.8 ± 0.22	114.96 ± 1.16	NA
GE-Au-2	100.6 ± 0.17	111.92 ± 0.309	137.26 ± 0.41
GE-Au-3	116.52 ± 15.55	90.47 ± 0.34	47.56 ± 0.21
NSE-Au-1	111.73 ± 0.6	76.81 ± 0.34	118.76 ± 0.86
NSE-Au-2	112.13 ± 0.77	NA	110.53 ± 0.26
NSE-Au-3	99.36 ± 0.1	115.53 ± 0.17	42.6 ± 0.11

## Data Availability

Data contained within the article and Supplementary File.

## Supplementary Materials

The following are available online at https://www.mdpi.com/article/10.3390/ma14123368/s1.
