# Supplementary material for: Evaluation of the Anticancer Activity of Phytomolecules Conjugated Gold Nanoparticles Synthesized by Aqueous Extracts of Zingiber officinale (Ginger) and Nigella sativa L. Seeds (Black Cumin)
Source: Materials (Basel). 2021 Jun 18;14(12):3368. doi: 10.3390/ma14123368 (PMC8234714; doi:10.3390/ma14123368)
Supplement: Supplementary file 1 [file materials-14-03368-s001.zip › materials-1222218-supplementary.pdf]

Supplementary Information

Evaluation of the Anticancer Activity of Phytomolecules Conjugated Gold Nanoparticles Synthesized by Aqueous Extracts of *Zingiber officinale* (ginger) and *Nigella sativa L.* seeds (Black Cumin)

Alaa H. Alkhathlan[1], Hessa Al-Abdulkarim[1], Merajuddin Khan[1], Mujeeb Khan[1], Musaed Alkholief[2, 3], Aws Alshamsan[2, 3], Aliyah Almomen[3, 4], Norah Albekairi[5], Hamad Z. Alkhathlan[1*] and M Rafiq H Siddiqui[1*]

1. Department of Chemistry, College of Science, King Saud University, Riyadh 11451, Saudi Arabia
2. Department of Pharmaceutics, College of Pharmacy, King Saud University, Riyadh 14511, Saudi Arabia
3. Nanobiotechnology Unit, College of Pharmacy, King Saud University, Riyadh 14511, Saudi Arabia
4. Department of Pharmaceutical Chemistry, College of Pharmacy, King Saud University, Riyadh Saudi Arabia
5. Department of Pharmacology and Toxicology, College of Pharmacy, King Saud University, Riyadh Saudi Arabia

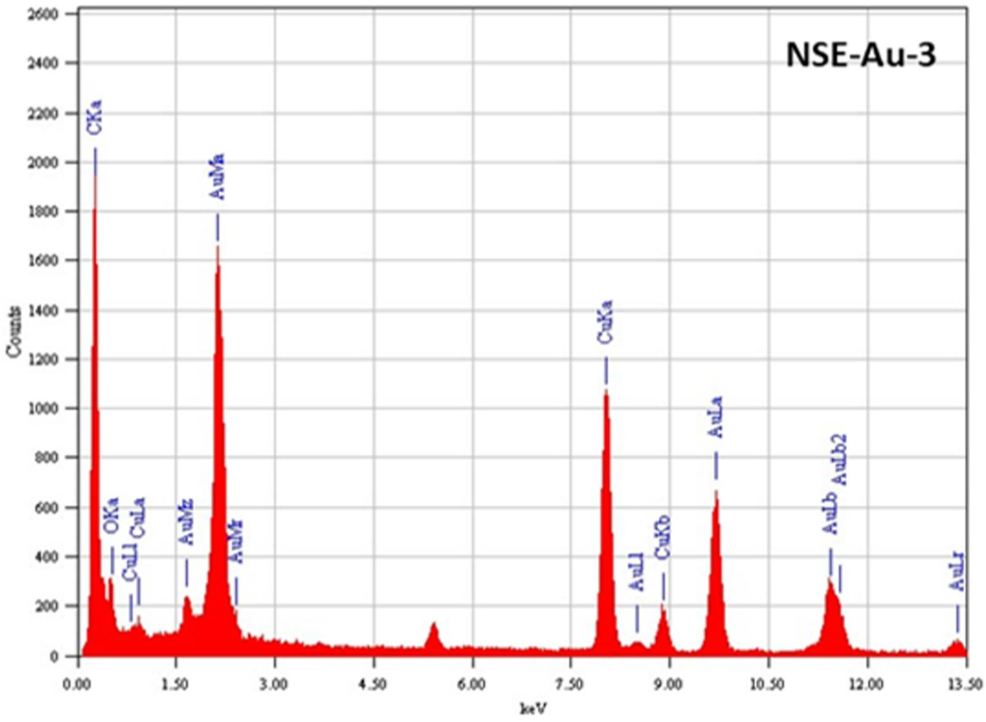

Figure-S1: EDS spectra of gold nanoparticles (Au NPs) prepared by using 20 ml NSE extract.

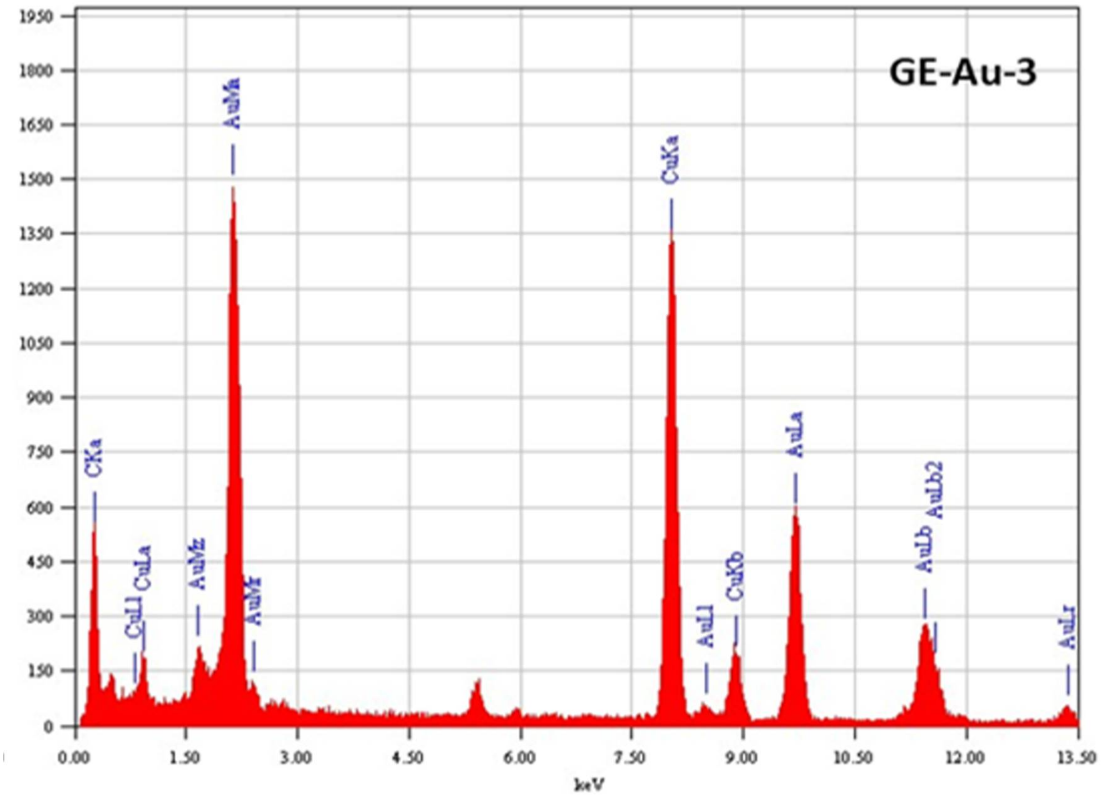

Figure-S2: EDS spectra of gold nanoparticles (Au NPs) prepared by using 20 ml GE extract.

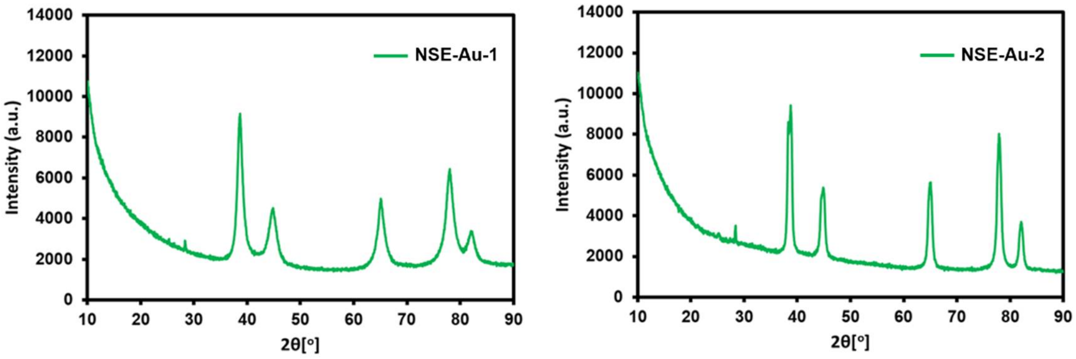

Figure S3: XRD pattern of gold nanoparticles (Au NPs) prepared by using low concentration of NSE, NSE-Au-1 by 5 ml and NSE-Au-2 by 10 ml of NSE extract.

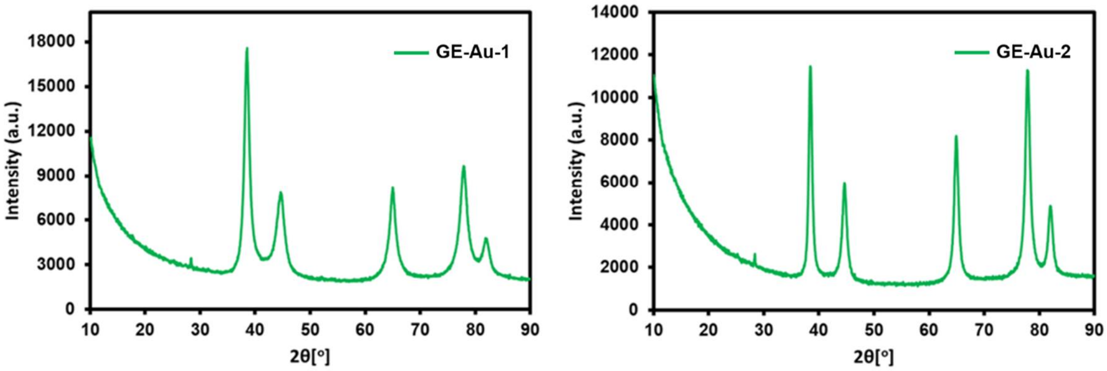

Figure S4: XRD pattern of gold nanoparticles (Au NPs) prepared by using low concentration of GE, GE-Au-1 by 5 ml of GE and GE-Au-2 by 10 ml of GE extract.
